# The Largest Liberty

**Published:** 1850-10

**Authors:** 


### THE LARGEST LIBERTY.

The radical politicians of the day have in much vogue among them the elastic phrase we have adopted as the caption of these remarks, and we cannot say that we feel much surprise in finding that the spirit, at least, of the phrase has crept into the medical politics of an establishment called the Memphis Medical Institute. The Trustees of that institution, with an admirable conception of the responsibilities of the trust bestowed upon them, have recently passed the following catholic views of medical teaching:

"Resolved, That this school of medicine is not to go under any sectarian or peculiar denomination; is not to teach any peculiar system of medicine; but all that is believed will throw light on the nature of disease, or contribute to its alleviation or cure,—and that it professes to be orthodox.

"Resolved, That each Chair will be expected to inculcate the doctrines which its Professor holds to be scientific truth; and that Professor Cross will teach the Institutes of Medicines as his convictions present, past, and future shall deem sanctioned by the light of science, and his own high reputation and exalted rank in the profession, and as most conducive to the well being and character of the Memphis Institute. To each Professor should belong the right to teach his own views and opinions connected with the branch of Medical Science committed to his Chair.

"Resolved, That in creating the new chair, over which Profestor W. Byrd Powell is to preside, it shall be designated as that of Cerebral Physiology, Mineralogy, and Geology, and in which Professor Powell shall have the privilege of treating of the external senses, and of so much of the nervous system in general, as he may deem requisite to a proper understanding of the functions and pathology of the brain."

If the Memphis Institute does not flourish like a green bay tree, under the auspices of such liberal concessions on the part of the Trustees to the Faculty, the Governors may feel assured that the fault does not lie at their door. The liberality extended to Professor Cross would seem to be the *ultima thule* in that line, until the reader finds himself involved in the labyrinths of the Professor Byrd Powell resolution. The beautiful harmonies, the Mosaic work of that gentleman's chair, that etherial body of light, known as Cerebral Physiology, surrounded with the tesselated border of Mineralogy and Geology, needed the expounding abilities of the trustees, and we are free to confess they have reached the usual result of commentaries,—by making confusion worse confounded. We had a dim, shadowy idea of Professor Byrd Powell's chair until we read the exposition by the trustees—from that moment, we realised the sensation of darkuess that may be felt. The trustees owe it to themselves, and to the institution over which they preside, to swing out, in front of their Medical edifice, a sign announcing "entertainment for all kinds of travellers." B.

